# Perception of childbirth experiences of Japanese women in Bali, Indonesia: a qualitative study

**DOI:** 10.1186/s12884-020-03466-x

**Published:** 2020-12-07

**Authors:** Kazuko Tanaka, Ni Made Dian Kurniasari, Desak Nyoman Widyanthini, Ni Luh Putu Suariyani, Rina Listyowati, Akimi Urayama, I. Made Ady Wirawan, Koichi Yoshimura

**Affiliations:** 1 Division of Midwifery, Yamaguchi Prefectural University, 6-2-1, Sakurabatake, Yamaguchi, 753-0021 Japan; 2 Faculty of Nursing and Human Nutrition, Department of Nursing, Yamaguchi Prefectural University, 6-2-1, Sakurabatake, Yamaguchi, 753-0021 Japan; 3 School of Public Health, Udayana University, Jl. PB Sudirman, Denpasar, Bali Indonesia; 4 Graduate School of Health and Welfare, Yamaguchi Prefectural University, 6-2-1, Sakurabatake, Yamaguchi, 753-0021 Japan

**Keywords:** Childbirth experiences, Japanese, Pregnant women, Bali Indonesia, Midwifery, Qualitative study, Woman-centred care

## Abstract

**Background:**

Maternal healthcare services in Indonesia have seen dramatic improvements over the past 25 years and yet there is still room for improvement. The perception, by the women, of the perinatal care provided, is a vital input to further improving these services. This study examines how the perinatal care provided is experienced by Japanese women in Bali, using an interview survey.

**Methods:**

We conducted semi-structured interviews, from August to October 2017, with 14 Japanese women living in Badung Regency and Denpasar City in Bali Province, Indonesia to report their perception of the perinatal care they experienced during their pregnancies. The interview guide included among others, the reasons for choosing specific (perinatal care) health facilities and their satisfaction with their experience of using the antenatal, delivery, and postnatal care services. The data were analysed using the qualitative content analysis method.

**Results:**

From the interview data, 12 categories across five themes were extracted. Participants reported experiencing various concerns during their pregnancies such as difficulty in obtaining perinatal care related information. From the beginning of their pregnancies, participants gradually established trusting relationships with midwives, but in many situations, they were disappointed with their childbirth experiences, as they felt that the care provided was not woman-centred. Through their own efforts and with the support of family members and other Japanese residents, many women were able to eventually regard their childbirth experiences as positive. Nevertheless, some women could not overcome their negative impressions even years after childbirth.

**Conclusions:**

Participants desired close attention and encouragement from nurses and midwives. Our results suggest that Japanese women in Bali expected a woman-centred perinatal care and active support from nursing/midwifery staff during their pregnancies and postnatal care.

**Supplementary Information:**

The online version contains supplementary material available at 10.1186/s12884-020-03466-x.

## Background

Indonesia’s maternal mortality ratio was 446 per 100,000 live births in 1990 however, as a result of government efforts to improve maternal health services, by 2015 this ration had fallen to approximately 126 per 100,000 live births [1]. However, perinatal care including the maternal mortality ratio in Indonesia still has room for improvement. Previous Indonesian studies indicated that midwives did not have enough clinical competencies and skills particularly in anaemia management during pregnancy [2] and that many pregnant women were unsatisfied with antenatal services provided by midwives [3].

Patient satisfaction can be defined as patients’ judgements regarding the quality of care they have received [4]. Women assess the quality of perinatal care received based on their satisfaction with the services provided, thus influencing their utilization of the available health facilities [5]. Measuring women’s childbirth satisfaction is complex and multidimensional; however, determining women’s satisfaction provides essential and cost-effective feedback that contributes to improving institutional childbirth services [6]. In the current study, we focused on assessing womens’ satisfaction with quality of care given by midwives as described in the document “International Confederation of Midwives (ICM) Essential Competencies for Midwifery Practice” [7]. A midwife is described as a responsible and accountable professional who works in partnership with women and gives the necessary support, care and advice during pregnancy, labour and the postpartum period, to conduct births on the midwife’s own responsibility and to provide care for the newborn and the infant [8].

All women, including Japanese, generally request and expect support from midwives during pregnancy, birth, and the postpartum period [9–13]. Many foreigners live in Indonesia, and according to Japan’s Ministry of Foreign Affairs, the number of Japanese residents in Indonesia is increasing, particularly in Bali [14]. The Japanese community in Bali is demographically young and is experiencing a baby boom [15]; these residents are predominantly women in their 30s and 40s living there with their husbands and children. Migrants around the world generally tend to have negative experiences with maternal care in their new countries. Previous studies have reported that migrant women in developing countries usually do not receive timely, appropriate or high-quality maternal health care [16–18]. A systematic and comparative review of immigrant and non-immigrant women’s experiences of maternity care, revealed that immigrant women were less positive about the care they received than non-immigrant women because of reasons such as communication problems and lack of familiarity with care systems [19]. Communication problems and lack of familiarity with health care systems had a negative impact on immigrant women’s experiences, as did perceptions of lack of respect, kindness and discrimination in the care their received, [19]. Non-native and immigrant women, including the Japanese living in Indonesia may have differing expectations and perceptions of the maternal care during their pregnancy and, as a result, they may be raising new issues and concerns that could be considered for further improving maternal and nursing care in Bali, Indonesia.

There is little research in Indonesia that has focused on maternal satisfaction, expectations or experiences of native and migrant women during the perinatal period. In our study we intended to explore, through respondent interviews, the perinatal experiences of the migrant Japanese women residing in Bali. Because many of these women could compare perinatal care in Indonesia with what is provided in Japan, we expected that they would identify more satisfaction and experience related issues than the local Balinese women.

## Methods

### Design

Our study employed a qualitative descriptive design using in-depth semi-structured interviews. Qualitative description represents the methodological category with the least level of inference among the qualitative methods [20], and in-depth semi-structured interview data constitutes the empirical backbone of qualitative research in the social sciences [21]. The theoretical framework that guided this study was a derived Donabedian’s model [22]. According to this model, quality of care is drawn from three categories: structure, process, and outcome. We used this model to understand childbirth experience and evaluate midwives’ quality of care, which allowed insight into mothers’ satisfaction during the perinatal period. To assess the quality of midwifery care we also referred to Halldorsdottir and Karlsdottir’s theory [23] and the concept of woman-centred care [24]. Halldorsdottir and Karlsdottir highlight five principal factors in a midwife’s profession. These five principal factors are: professional caring, competence and wisdom, empowering interaction and partnership together with the midwife’s personal and professional development holistically combined [23]. In order to focus our objective, we asked participants about their childbirth experiences, their satisfaction or dissatisfaction with the attitudes of, and services provided by midwives during the perinatal period. The consolidated criteria for reporting qualitative research (COREQ) checklist was used to guide reporting of this study. The COREQ checklist contains 32 items [25].

Qualitative research is based on interpretation, which requires input from researchers. Although interpretation is subjective, the authors of this study are well qualified to understand the nuances of the topic. The first author (KT) is a female Japanese midwifery lecturer in a university who has had midwifery experience in Japan and Southeast Asia. From her personal experience, the first author felt there was room for improvement in perinatal care in Southeast Asia, especially in midwifery care during birth. All Indonesian co-authors are public health and maternal and child health research experts (NMDK, DNW, NLPS, RL, IMAW) and university lecturers. As for the Japanese co-authors, AU is a female professor in the division of midwifery and KY is a male medical doctor and professor in the Graduate School.

### Setting

Bali is renowned worldwide as a popular tourist destination and has many foreign residents and immigrants from other islands in Indonesia. Bali is one of the most developed islands in Indonesia in terms of economic activity, infrastructure, and population [26]. The number of Japanese residents in Bali is nearly 40 times greater than it was 30 years ago. At the time of this study, a Japanese Foreign Ministry survey showed that there were approximately 3000 Japanese nationals residing in Bali [14]. The data for this research were collected from the Badung Regency and Denpasar City (the province’s capital) in Bali Province, where three quarters of these Japanese residents in Bali were located [14].

### Sample recruitment

For the purpose of this study, Japanese women, aged 26–42 years, who had given birth in Bali, were recruited. Every woman who consented to participate in the study had to meet the criteria of having their youngest child to be under five. All of these women had knowledge of and many had experienced maternity care services in Japan. Only 3 women from these recruited women had never given birth before. Nevertheless, based on their knowledge, the authors concluded that they could still compare their perinatal experiences in Indonesia with those in Japan. The first participant was introduced to the researchers by a Japanese permanent resident working in Bali. Through the snowball sampling method, a total of only 14 participants were recruited. The researchers collected data between August and October 2017.

### Data collection

The quality of midwifery services plays a decisive role in a woman’s experience of childbirth [23], and because recent work in policy and service provision surrounding maternity care recommends a woman-centred approach [27, 28], we developed an interview guide for this study based on the notion of a midwife’s professionalism [23] linked to the concept of woman-centred care [24]. The Japanese researchers (KT, AU, KY) designed the first draft of the interview guide and then consulted about it with Indonesian researchers (NMDK, DNW, NLPS, IMAW) (Additional file 1). The interview guide was tested on the local Balinese women to gain insight into what sort of satisfaction and services related issues that may be raised by these women using these interview questions. Coincidentally, there was similar research being conducted at the same time on Balinese women by our research group and so we decided to use the same interview guide for interviewing the Japanese in light of the limited time we had for the study. Their answers, we concluded, could provide insights that would assist the local and Japanese interviewers to look at any similarities and differences in their responses and types of issues raised.

Semi-structured, face-to-face interviews were conducted with the 14 participants to explore their explanations, perceptions and experiences with the perinatal midwifery care they received during their childbirth experiences. Before starting the interviews, consent and background information was obtained, including age, religion and duration of stay in Bali, length of the marriage, reproductive history, and Indonesian-language proficiency (Additional file 2). In our interview guide we included questions on reasons for choosing a particular childbirth facility and their explanations, perceptions and experiences (both positive and negative) about their midwives’ attitudes and services they received during the perinatal period, including their knowledge and skills, interpersonal communication abilities, partnering with women and their families, and so on (Additional file 1). All of the participating women consented to being interviewed which were conducted in the participants’ homes or at other mutually agreed places where privacy could be guaranteed. The interviews were audio recorded and the participants’ consent and contemporaneous notes were also taken.

The first author (KT) conducted the semi-structured face-to-face interviews in Japanese. The Indonesian researchers also participated in the interviews taking field notes with the support of a professional female Japanese interpreter who was familiar with local medical terminologies and conditions. The interpreter received advance orientation about the aim of the study and its themes. Each interview was jointly conducted in Japanese by the first author and an Indonesian researcher supported by the interpreter to enable multiple observations and conclusions. Each interview lasted approximately one hour. At the end of the interview, the interviewer (KT) verbally summarized the key points and asked the participant if the summary was accurate. Data collection was characterised by openness to new ideas among the interviewers and reinforced by the follow-up probing questions during these interviews. Theoretical saturation was achieved after 14 interviews as no more new information emerged.

### Data analysis

The data were independently analysed using the qualitative content analysis in accordance with Graneheim and Lundman’s methods [29]. Content analysis is defined as a research technique for identifying replicable and valid inferences from data in context [30]; it is a flexible method for analysing textual data [31]. This method can be useful as one stage of data analysis because it allows the relevance of a pre-existing theory to be tested, and it can be used as a way of assessing the applicability of theory [32]. The process includes open coding, category creation, and abstraction [33].

After completing the interviews, the Indonesian and Japanese researchers (KT and NMDK, DNW, or NLPS) involved in conducting the interviews reviewed and discussed the childbirth experiences that had been described by the participants. Recordings were transcribed verbatim into Japanese by the first author and later translated into English by one professional translator. We used only one translator to ensure the reliability of the data [34]. English transcripts were given to the Indonesian researchers (NMDK, DNW, NLPS) who checked it and compared it with their field notes.

The data analysis process was led by the Japanese researchers (KT, AU, KY). All Japanese researchers and Indonesian researchers (NMDK, DNW, NLPS) repeatedly read the verbatim records in order to familiarise themselves with the participants’ expressions, and then extracted and summarised related text in order to avoid changing the individual participants’ overall meanings. Researchers performed the analysis separately and then discussed the results as a team and arrived at a consensus [30, 35]. The five phases of analysis [33] began with the researchers familiarising themselves with the data and identifying the preliminary codes. The codes were integrated into subcategories until the main categories emerged. This process allowed us to identify key comprehensive themes that described the investigated phenomena, as well as the key elements of interest. We developed categories and themes, then compared the categories and themes generated from the Japanese and English data sets [34]. Categories and themes were refined, reviewed and reconciled going back to the data repeatedly to ensure consistency. Triangulation was used to reduce the effect of research bias to establish confirmability, including reflexive field notes. Interview data, data analysis products, and data reconstruction products were provided to the research team for verification. To ensure reliability, field notes and individual interview data were compared to intensively examine the consistency of data [36, 37]. The ATLAS.ti 8 software package was used to support analysis of the transcripts.

### Ethical considerations

Researchers adhered to the Declaration of Helsinki recommendations in conducting this research. Ethical approval was obtained from the Faculty of Medicine, Udayana University/Sanglah Hospital (493/UN.14.2/KEP/2017), and Yamaguchi Prefectural University (No.27–45). Before data collection, the purpose and aim of the research were explained to the participants, and they were informed about their rights. Written and verbal information was given to participants, and written consent was obtained before the interview. Participants were also assured that all their responses would be kept confidential; all collected information including audio recordings and transcripts were securely stored and accessible only to the research team. This study is a part of an ongoing project (2016–2021), which was implemented in cooperation with Udayana University and Yamaguchi Prefectural University.

## Results

### Participant characteristics

The participants’ average age was 37.6 (± 4.7) years. Of the 14 participants, three had a high-school education and 11 had tertiary education. The average length of stay in Bali was 8.8 (± 5.1) years; the majority 10(71%) had been in Bali for over five years. Most of the women’s husbands were Indonesian. {10(71%) were Indonesian, 4(29%) were Japanese}.

Six participants were Muslims, three were Hindu, (they were required to convert when they got married with Indonesian men) and five were adherents of other religions. Over half of the participants could read and write in Indonesian.

Of the 14 participants, 11 had given birth two or more times, and six of the 14 women had also experienced childbirth in Japan. In terms of the choice of birthing facilities, 11 participants had chosen private hospitals and three had chosen a midwives’ clinic (*bidan* in Indonesian). Six women had given birth vaginally, two had been induced, five had had caesarean sections, and one had experienced a waterbirth. All babies were full-term. The average age of their youngest child was 21 (± 15) months. Only three participants possessed the Indonesian government’s Mother and Child Health (MCH) Handbook, while over half of the women retained the Japanese government’s MCH Handbook (Table 1).

**Table 1:** Characteristics of participants

Variable	*N* = 14 (%)
Age: mean (SD)	37.6(±4.7)
Length of stay in Bali: mean (SD)	8.8 (±5.1) years
<5 years	4 (28)
5–10 years	5 (36)
>10 years	5 (36)
Length of marriage: mean (SD)	9.1 (±5.8) years
<5 years	3 (21)
5–10 years	6 (43)
>10 years	5 (36)
Age of husband: mean (SD)	39.3 (±6.1)
Religion	
Muslim	6 (43)
Hindu	3 (21)
Other	5 (36)
Educational background	
High school education	3 (21)
Tertiary education	11 (79)
Age in months of last child: mean (SD)	21 (±15)
<12 months	3 (21)
12–24 months	6 (43)
> 24 months	5 (36)
Partner characteristics	
Indonesian	10 (71)
Japanese	4 (29)
Parity	
1	3 (21)
2	9 (65)
3	1 (7)
5	1 (7)
Place of birth	
Private hospital	11 (79)
Midwives’ clinic (bidan)	3 (21)
Mode of birth in Bali	
Normal	6 (43)
C-section	5 (36)
Indonesian speaking ability	
Low	3 (21)
Basic	8 (58)
Good	3 (21)
Indonesian reading/writing ability	
Low	5 (36)
Basic	7 (50)
Good	2 (14)
Indonesian Maternal and Child Health Handbook	
Yes	3 (21)
No	11 (79)
Japanese Maternal and Child Health Handbook	
Yes	9 (64)
No	5 (36)

### Themes and categories

**Fig. 1:**
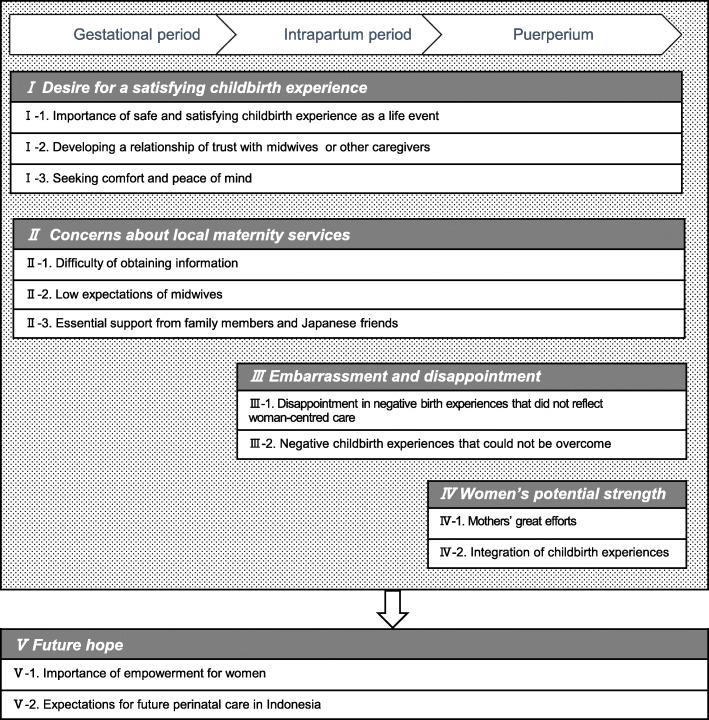
Themes and categories of the childbirth experiences of Japanese women in Bali. Note: Themes are in italics and categories are listed below the themes

We identified 12 categories across five themes, which are described below (Fig. 1). During their perinatal period, the participants strongly emphasised safe and satisfying childbirth experiences (Fig. 1: I-1). However, they had various worries and concerns regarding issues such as the difficulty of obtaining information pertaining to pregnancy and childbirth (Fig. 1: II-1). For this reason, support from family members and Japanese friends who had previously worked as midwives and nurses in Japan, was essential from the beginning of their pregnancy (Fig. 1: II-3). Although the participants did not have high expectations of the midwives caring for them (Fig. 1: II-2), they did seek comfort and peace of mind (Fig. 1: I-3). From the beginning of their pregnancies, they gradually built relationships of trust with the midwives or other caregivers (Fig. 1: I-2), but many were disappointed with their childbirth experiences, as they felt that the care provided during labour was not centred on the woman giving birth (Fig. 1: III-1). However, through both their own efforts (Fig. 1: IV-1) and with the support of family members and the Japanese friends as mentioned earlier (Fig. 1: II-3), many women were able to reflect on their childbirth experience positively (Fig. 1: IV-2). However, some women could not achieve this, even years after they had given birth (Fig. 1: III-2). Additionally, empowerment of mothers during pregnancy, childbirth, and the postpartum confinement was regarded as important (Fig. 1: V-1), and participants expressed their hope that perinatal care in Bali, Indonesia would further improve in the future (Fig. 1: V-2).

#### Desire for a satisfying childbirth experience

When the participants were preparing to give birth in Bali, they desired a safe pregnancy and childbirth experience. They had a variety of concerns and worries regarding giving birth at an older age or fears of miscarriage and unpredictability in the onset of labour, and they wanted a high level of maternity care. Consequently, they placed high importance on finding specialist obstetricians and well-equipped health facilities where they could undergo maternity related ultrasound examinations. These women also preferred natural childbirth, to give birth with their husbands in attendance, doula support, kangaroo mother care, and/or exclusive breastfeeding. As a result, they selected childbirth facilities that could provide these services. In effect, these women were expressing the sort of childbirth plans they preferred.

*A Japanese friend who had been a midwifery professional said that she would be present for the birth, even if it was in the middle of the night. Also, I kept hearing that hospitals here perform a lot of caesarean sections, so I asked them to perform a caesarean section only if the situation became very bad and with my consent, but, if there were no problems, I said that I would prefer to have natural childbirth. The doctor said that it was his policy was to proceed with a natural childbirth whenever possible (JPN5).*

After becoming pregnant, the Japanese women searched for an obstetrician they could trust, underwent regular health check-ups, and then gave birth at the hospital or clinic where their attending obstetrician worked. The women who chose to give birth in midwives’ clinics reported developing trust in their midwives because of the midwives’ attitudes, good listening skills, and their efforts to help the birth occur in a home-like atmosphere, and also because they provided advance explanations of the procedures that were to be undertaken.

*The (bidan) staff were wonderful people. They always smiled when they spoke, and if I had diarrhoea when I visited, they would give me lots of advice [ … ], so I kind of depended on them (JPN6).*

Almost three-quarters (71%) of the Japanese women’s spouses were Indonesian, and they were based in Bali; consequently, they chose to give birth in Bali rather than travel back to Japan despite their anxiety. Some women sought a feeling of religious solidarity and chose care providers of the same religious faith, while others sought a sense of security and peace of mind at accessible midwives’ clinics (*bidan* in Indonesian). Women with no experience of childbirth found greater peace of mind in giving birth in familiar surroundings. Moreover, they were grateful that they could, without feeling guilt or embarrassment, ask staff to look after their infants after birth, a common practice in Bali, unlike in Japan.

*I heard in Japan that if you asked hospital staff to look after the baby at night, they would make a stern face, as if to say: ‘No, you’re the mother, you look after it’. Friends told me that staff would refuse to look after the baby, saying things like, ‘It’s born now; that is the mother’s job’, but in Indonesia, this isn’t the case. I’ve heard that in Japan, the nurses make you feel really guilty (JPN5).*

#### Concerns about local maternity services

Even participants who had no difficulty engaging in everyday conversations in Indonesian had some trouble communicating with midwives, as a result of unfamiliarity with specialist terminology, and many participants reported receiving support from Japanese friends who had also been midwives and other similar health-care professionals who worked in Indonesia. The women experienced difficulty obtaining information related to local maternal and child health services and facilities because of language and technical barriers and were worried that, as foreigners, they could be at a disadvantage. They feared that they would undergo an unnecessary caesarean section without their consent.

Three women were given the Indonesian government’s MCH handbook. Indonesia’s maternal care is unlike Japan’s [2, 38], where fourteen maternity check-ups are provided without charge. As this was not the case in Bali, participants felt they had less support provided to them.

Consequently, the local maternity care system added to the participants’ unease regarding giving birth and other pregnancy-related difficulties. The women also felt anxious about the fewer number of examinations conducted during pregnancy in Bali when compared to Japan, and they had doubts regarding diagnoses and prescriptions. Additional differences in the maternity services between Bali and Japan also caused great concern, such as the short duration of postpartum hospital stays, generally one night and two days [39, 40]; when compared with the postpartum hospitalisation period in Japan of 5–7 days [41, 42].

*They have many caesareans here. I would hate to have a caesarean. I wanted, if possible, to give birth naturally. Here, there is a tendency to quickly resort to surgery. All of the staff at my company gave birth via caesarean section (JPN3).*

*I would like them to carry out a few more tests such as urine tests and blood pressure tests before determining that a C section was really necessary for the baby’s and the mother’s sake, (JPN9).*

They had adopted an attitude of generally not having high expectations during their social inter- personal encounters and this also extended to health staff, meaning they had fewer expectations from midwives from the beginning. Consequently, even when the level of care they received differed from what they hoped for, they convinced themselves that it could not be helped.

*Having no expectations for maternity care is the best way not to get hurt, and my attitude is the same toward some of the other local people. After living here for seven years, you get good at it. But don’t get me wrong, Indonesian people are good people; I love them (JPN3).*

Many of the participants had networks of Japanese people they could consult. They were aware of the importance of support from their family and Japanese friends who had been midwives and who also worked in Indonesia. When they, the respondents gave birth, their mothers arrived from Japan to assist them, and Japanese friends who had been midwives living in Indonesia provided support until the postpartum confinement period ended.

For women with Indonesian husbands, there was, on one hand, the added hardship of receiving a large number of relatives who visited after the birth but, on the other hand, family members also provided essential assistance, such as support for breastfeeding and various other aspects of child-raising.

*Ms. A. (Japanese friend who had been a midwife) gave me detailed explanations, and I was able to consult Ms. A. about even trivial matters. She was that kind of person. Accordingly, I was not anxious in the least, and it was helpful that she stayed with me during the birth as well (JPN2).*

#### Embarrassment and disappointment

During pregnancy check-ups in hospital by the obstetricians, the Japanese women had virtually no interaction with midwives. Having the belief that health guidance comes from the midwives, based on their knowledge of Japan, this lack of health guidance made them feel anxious. During childbirth, doctors directly assisted with the labour; so the women did not remember any nurses or midwives being present in the birthing room. Comparing this with the situation in Japan, where midwives help during labour, it was understandable the women sensed the extensive authority of the physicians and felt that midwives had an unclear role. Because of this comparison, they were especially discomfited by light level of midwifery and nursing care during labour and birth. When the women entered the hospital with labour pains or after their membrane ruptured midwives checked on them in accordance with their local standards and, since they did not receive clear advice regarding alleviating childbirth pain or actions they should take during labour when compared to their knowledge and experience in Japan, participants felt abandoned and also experienced distress because they perceived that they did not receive the expected support during labour.

Many participants were especially anxious about breastfeeding, unsure about how to treat the umbilical cord when bathing the baby, and they expressed concern about limited guidance provided. Even when the women had the support of family members or friends, they still expected additional health based guidance from professionals. Despite planning for a vaginal birth, women did not feel that they were given enough support from the midwives regarding education on pain management during birth, guidance on breastfeeding, bathing the baby, and so on. Women who had previously experienced childbirth in Japan perceived significant differences in maternity care provided in Bali when compared to Japan that became a source of concern for them.

*The most memorable episode was that when I was giving birth in Japan to my first child, a midwife supported me during labour to avoid push with each contraction, but I didn’t have such specific support here (in Bali) (JPN1).*

*If the staff had knowledge, I wish they had shared it with me. Rather than leaving the breastfeeding process after birth to the mother, I would have liked it if they had provided a little more guidance. … I thought that they probably don’t provide guidance for how to bathe the baby and every little thing because there are family members to do that (JPN9).*

Those experiences left women dissatisfied with their experiences during and after birth, and hoped they could have better experiences in the future. Women who were unable to bear the labour pains and underwent caesarean sections felt regret that they could have perhaps borne the pain if they had received help and encouragement.

*I was in the throes of birth, I couldn’t cope with labour pain, I asked help from the midwife, but she just said ‘Not yet’. I felt increasingly sad and neglected because no one did anything for me during labour. Finally, I said, ‘Please perform caesarean’ (JPN8).*

#### Women’s potential strength

After giving birth, many of the participants did not receive clear guidance about breastfeeding or caring for the infant and consequently taught themselves instead. This is the way it is culturally done in Bali because of the available family support structures. While they were in hospital, they watched internet videos and referred to Japanese leaflets and childcare books since many of these were in Japanese and therefore understandable. Furthermore, those who had experienced childbirth in Japan drew on their previous and existing knowledge of labour and child-rearing to use in their situation.

*They didn’t teach me anything about how to care for the baby, even how to change a diaper. [ … ] I didn’t really expect the hospital staff to teach me these things, and so I read various (Japanese) books on child-raising. (JPN7).*

Even though some of the women were dissatisfied with various aspects of the perinatal care they received, they were relieved that their babies had been born healthy. They did not regret their decision to give birth in Bali and were able to regard their pregnancy and labour experiences as positive.

*I had really strong feelings about ‘What if it had been a natural birth’? but all of a sudden they decided I needed a caesarean and I just said something like, ‘OK, as long as the baby is born healthy’. Ultimately, I felt that it was more important that the baby was born healthy than whether I wanted to do this or that. And so, in the end, I was just happy that the baby was born healthy, and that we were able to leave the hospital and that the baby grew healthily (JPN11).*

#### Future hope

At Japanese childbirth facilities, healthcare guidance is provided through mothering classes and individual instruction from midwives, but only a few of the participants received similar guidance in Bali. Many of the women studied these things on their own, using the internet and other resources. During pregnancy check-ups, they felt that information and explanations were not actively being provided to expectant and nursing mothers; for instance, regarding examination results, they were simply told if there were any problems but they were not given additional detailed information to reassure the mother. The women also reported that information was not offered on topics such as weight gain and foods to be avoided during pregnancy. The participants knew that excessive weight gain during pregnancy can increase the risk of gestational diabetes, macrosomia, and/or hypertensive disorders of pregnancy, and they felt that they needed to take the initiative to obtain relevant information. This was probably due to the fact that the advice given to the local Balinese pregnant may not be appropriate for the Japanese participants. The participants also thought that the care and limited information provided to the local expectant mothers by their doctors who were in charge of their maternity care as problematic when compared to their knowledge and experiences in Japan. This highlighted the need for childbirth preparation and education for foreign expectant women that can help broadens their options.

*My hands and feet were really swollen and puffy; my blood pressure was elevated. I was worried, so I did a search on the Internet, and I found quite a number of diseases/conditions I would probably have been diagnosed with in Japan, such as hypertensive disorders of pregnancy. That was my situation, but here I didn’t receive any particular explanation or advice (JPN5).*

In contrast to Japan, Indonesia has a high birth rate. Some participants thought that their obstetricians and gynaecologists would have a great deal of experience, and that there were also veteran midwives who had established their own practices. The participants wanted to receive care from experienced professionals. Some participants who experienced discomfort during pregnancy found relief after receiving treatment at midwives’ clinics, while other participants were pleased to receive breast massages after birth, which improved milk production.

*When I went to my husband’s parents’ after childbirth, I got breast massage from an elderly traditional midwife, it helped me with exclusive breast-feeding. There are many midwives’ clinics here, they shared with us useful pieces of maternal health trivia, and they were friendly (JPN10).*

*It may be that in Indonesia having children is almost too routine; pregnancy and childbirth are not treated as big and special events. But I think the process could be made a little more enjoyable. As a mother, there are only a limited number of times you can experience childbirth in your lifetime, aren’t there? That’s why I think there could be more opportunities for enjoying the childbirth process (JPN7).*

*I think the care provided for childbirth is OK as far as locals are concerned, but I think that health-care professionals at health facilities where foreigners are treated need to provide more information and support about postpartum care and caring for pregnant women. (JPN13)*

## Discussion

### Need for information

Private hospitals are the most popular maternity facilities in Bali and Indonesia (used by 48% of women [26];), and the substantial majority of our participants gave birth in private hospitals in Bali. When choosing a facility for prenatal check-ups and childbirth, the participants consulted their networks of Japanese people as well as local information. For foreign mothers generally, maternal and child health information in their native language is extremely limited and among our participants, few possessed the Indonesian version of the MCH Handbook which was adapted from Japan’s maternal and child health system [43]. The Handbook facilitates the continuum of care throughout pregnancy, birth, postpartum, and the child’s infancy, and thus has the potential to be an effective tool for improving health awareness and client-provider communication. It includes maternal and child health tools that record the maternity related information as well as child development milestones from 0 to 5 years [44]. However, in Bali, Indonesia, there were some private health facilities where no MCH Handbook was available. These were found mostly at government-owned public health centres. Thus improving the availability of the local MCH Handbook in all maternity health care facilities available to all mothers would strengthen the maternity care services.

### Need for responsive and competent staff in the maternity care facilities

In 2012, the rate of caesarean sections in Indonesia was 12% [45] but, according to earlier research, there was a disparity regarding this between maternity facilities in urban and rural areas, with rates being higher in more urbanised areas such as Java and Bali [26]. Caesarean section rates are continuing to rise, particularly in high- and middle-income countries [46, 47]. When medically justified, a timely caesarean section can effectively reduce maternal morbidity and mortality; however, there is no evidence of the benefits of caesarean birth for women or infants who do not require the procedure [48]. According to new research by Indonesian researchers, the rate of caesarean sections is 23.0% in the urban areas of Indonesia [49].

All participants were aware of the high rate of caesarean sections in Bali; therefore, this was a major factor increasing their anxiety (as they preferred to give birth naturally). Even though the foetus and mother were not in danger, some participants requested caesarean sections during the first stage of labour because they were unable to bear the pain of childbirth. These women strongly believed that they would have been able to withstand the pain with active support, encouragement and explanations about their diagnoses from their midwives and other health staff.

Women who received continuous support during labour were more likely to give birth ‘spontaneously’; that is, vaginally without the use of ventouse, forceps, or a caesarean section. In addition, such women were also less likely to use pain medications and to feel more satisfied and have shorter labour [50]. Moreover, in Bali, with the support of their mothers and other family members, the women did their best to overcome their unsatisfactory experiences during childbirth.

Mental health issues can easily arise during the early postpartum period [42, 51, 52]; although the majority of women coped well. Nevertheless, for some, childbirth was a stressful experience; this is of concern given that in previous work it has been reported that childbirth-related stress increases the potential for developing postpartum posttraumatic stress disorder symptoms [53]. Literature reveals that the prevalence of postnatal depression is highest among migrant women [54]. Brief midwife-led counselling interventions for women who report distressing birth experiences have been found to be effective for reducing symptoms of trauma, depression, stress, and coping with feelings of self-blame [55].

### Woman-centred care

After childbirth, all new mothers tend review their childbirth experiences and events, reflecting on how they differed from what they had expected, and assimilate this experience in line with their expectations [56]. Recently, in Japan, there has been an increase in the number of maternity facilities where women who had just given birth along with midwives, can review their labour experience during the early postpartum period [57, 58]. Some of our study participants were disillusioned with their childbirth experiences and continued to recall this disillusionment during their interviews. For such women to overcome their negative childbirth experiences, reviewing their childbirth experiences with a midwife during the early postpartum period would have been an effective measure to help with coping better.

Over the past 20 years, there have been various studies mostly in UK and Japan on women-centred care during pregnancy and childbirth [24, 27, 28, 38, 59–62]. The qualitative and quantitative results of a previous survey of expectant and nursing mothers done in the UK indicated that there is a need for woman-centred care through which midwives form partnerships with expectant and nursing mothers, and help expectant and nursing mothers make information-based choices [63, 64]. Many of our participants were unable to form such partnerships with their local midwives during the perinatal period, consequently were unable to achieve the desired childbirth experience. Our results suggest that empowering women through the provision of information and attentive care throughout the perinatal period would be a valuable improvement. To enable women to approach pregnancy and childbirth proactively, and to have safe and satisfying childbirth experiences, it is essential that they receive support from their midwives that could contribute to further improvements in perinatal care in Bali, Indonesia.

### Limitations

In this study, only Japanese women living in Indonesia were selected as study participants because they could compare their experience of perinatal care provided both in their local area Bali and in Japan. This comparison could help reveal relevant issues relating to perinatal care by comparing maternity care services in Bali, Indonesia and Japan. Thus, women’s perceptions about their experiences are solely described from the viewpoint of Japanese women which limits the value of the comparison between their perception of the care in Bali Indonesia and their recollections of their care in Japan. In addition, our study participants all used private facilities for their maternity care during their perinatal period. In Indonesia, giving birth in private facilities has increased to 48.1%, but many other women still give birth in public health facilities and at home [26].

## Conclusions

This study shows that the perception of the childbirth process as experienced by Japanese women in Bali, Indonesia during the intrapartum and puerperium periods was generally not woman-centred. The selected Japanese women who were interviewed sought, but, in most cases, did not receive, active support and encouragement from midwives who they interacted with during their childbirth experiences. Our results highlight the need for providing a more woman-centred care approach that includes the empowerment of women generally and, Japanese women especially, during the perinatal/maternity period. In general terms, maternal health care in Indonesia has significantly and consistently improved over the past 25 years and further research from women’s perspectives, especially from foreign women, provide critical insights from the lessons learned, that could contribute to improving the quality of perinatal/maternal care in Bali in particular and, in Indonesia generally consistent with the evolving international standards, and yet responsive to local socio-cultural needs.

Women were seeking clear and detailed explanations and an accommodating attitude by midwives during perinatal/maternity period. Taking action to improve maternal care services by training and providing specific practice guidelines, adequate interpersonal communication skills and providing sufficient and effective skill development training and health education materials to be used for counselling is required. To develop appropriate information education and communication materials which are easy-to-understand even for foreigners could positively improve the health education provided by midwives. In addition, to contribute to improving maternal health services, it is necessary for medical professionals such as midwives to form partnerships with women, respect them, and cooperate with them. We conclude that midwives need to respect women’s choices so that women themselves can be enabled to face their pregnancy and childbirth constructively and safely and with reassurance, and build a system to help these women to take the initiative in childbirth.

## Supplementary Information

**Additional file 1.**

**Additional file 2.**

## Data Availability

The datasets used and/or analyzed in this study are available from the corresponding author on reasonable request.

## Abbreviations

ICM: International Confederation of Midwives
COREQ: Consolidated criteria for reporting qualitative research
MCH: Maternal and Child Health
UK: United Kingdom
