# Supplementary material for: Perception of childbirth experiences of Japanese women in Bali, Indonesia: a qualitative study
Source: BMC Pregnancy Childbirth. 2020 Dec 7;20:760. doi: 10.1186/s12884-020-03466-x (PMC7720464; doi:10.1186/s12884-020-03466-x)
Supplement: Supplementary file 1 — Additional file 1. [file 12884_2020_3466_MOESM1_ESM.docx]

**Interview Guide**

**Questions**

1. Please tell us about your previous childbirth experiences till now. Why did you choose this particular facility to give birth？
2. We would like to ask you about your pregnancy and delivery in Bali, Indonesia. (We will ask about the following points, and yet we make sure that the respondent is able to talk as freely and spontaneously as possible. We will then follow up by asking why the respondents marked satisfied or unsatisfied for the points noted below.

- Problems during pregnancy, delivery, and the postpartum period, and support obtained from midwives and other staff for dealing with these problems.
- Delivery: time required for delivery, condition of the child from the mother's point of view, birth abnormality/medical treatment at delivery, position at delivery, early mother-to-child contact, whether anyone witnessed the delivery, emergency response, and continuous care.

1. Could you describe how you felt about the attitudes and services provided by the midwives and other staff? What did you find satisfactory or unsatisfactory, and why? (We will cover the following main points, but will, also give full consideration and freedom and allow the respondent to speak as freely and spontaneously as possible.)

- Explanation and consent to medical treatment.
- Language used, attitude, and respectful attitude.
- Content of guidance provided, treatment, and instructions given by the midwives during pregnancy, delivery, and the postpartum period.
- Confidence in the midwives' diagnosis, their examination and the treatment during the process of childbirth.
- Assistance provided by the Midwives' to ensure that the birthing process would be in line with the woman's wishes, such as provision of massage and natural childbirth. (narrative-based medicine, respectful and courteous care for pregnant women and their families)

1. Reflecting on your pregnancy and childbirth experience in Bali, please tell us if you had any further expectations from midwives.
2. If you were to give birth in the future, what kind of birth experience would you like to have? Why?
3. If you were to give birth in the future, where would you like/prefer to give birth? Why?
