# Supplementary material for: Perception of childbirth experiences of Japanese women in Bali, Indonesia: a qualitative study
Source: BMC Pregnancy Childbirth. 2020 Dec 7;20:760. doi: 10.1186/s12884-020-03466-x (PMC7720464; doi:10.1186/s12884-020-03466-x)
Supplement: Supplementary file 2 — Additional file 2. [file 12884_2020_3466_MOESM2_ESM.docx]

**Face Sheet**

Date of interview

No.

Thank you for agreeing to participate in our research today.

Before we interview you, we would like to ask you a few questions. This information will be kept strictly confidential, and will not be used for any other purpose except in this research.

Please answer the questions as below by filling in the blanks or checking the appropriate box.

**Basics**

Age　　　　　　　　Occupation　　　　　　 　 　　　　　Religion

Highest educational qualification

Duration of stay in Bali　　　　　years

Nationality　　　　Japanese / Indonesian

If you are Japanese, do you plan to change your nationality?　　　　　　Yes / No

Period of marriage　　　　　　years

Age of husband　　　　　　years old　　　　Occupation of husband

Family members

**Reproductive history**

1. Date of delivery Sex of infant □male  □female

State of infant’s health □good 　 □asphyxia

Birth weight g Place of birth

Mode of delivery □normal □induced delivery　　 □vacuum extraction　　□C-section

1. Date of delivery Sex of infant □male □female

State of infant’s health □good 　 □asphyxia

Birth weight g Place of birth

Mode of delivery □normal □induced delivery　　 □vacuum extraction　　□C-section

1. Date of delivery Sex of infant □male □female

State of infant’s health □good 　 □asphyxia

Birth weight g Place of birth

Mode of delivery □normal □induced delivery　　 □vacuum extraction　　□C-section

1. Date of delivery Sex of infant □male □female

State of infant’s health □good 　 □asphyxia

Birth weight g Place of birth

Mode of delivery □normal □induced delivery　　 □vacuum extraction　　□C-section

**Other**

Do you have an Indonesian or Japanese version of the Maternal and Child Health handbook? Yes / No

Do you have a person in Bali, with whom you can discuss concerns about pregnancy, delivery, and the postpartum period?

　 Yes / No

Indonesian language ability (conversation)

□ Daily conversation is difficult.

□ I am worried that I will not be able to speak the local language when I take my child to the hospital without an escort who can speak the local language.

□ I can visit the hospital alone.

Indonesian language ability (reading and writing)

□ I can barely read it.

□ I can answer a medical questionnaire.

□ I can read newspapers.
